# Locked up at home: a cross-sectional study into the effects of COVID-19 lockdowns on domestic violence in households with children in Belgium

**DOI:** 10.1186/s12889-022-14135-3

**Published:** 2022-09-10

**Authors:** Elizaveta Fomenko, Lotte De Schrijver, Christophe Vandeviver, Ines Keygnaert

**Affiliations:** 1 ICRH – Faculty of Medicine & Health Sciences, Ghent University, Ghent, Belgium; 2 IRCP – Faculty of Law & Criminology, Ghent University, Ghent, Belgium

**Keywords:** Domestic violence, Children, COVID-19 pandemic, Public health, Sexual and gender based violence

## Abstract

**Background:**

Policymakers worldwide took measures to limit the spread of the COVID-19-virus. While these sanitary measures were necessary to fight the spread of the virus, several experts warned for a significant impact on mental health and a potential increase in domestic violence. To study the impact of the COVID-19 measures in Belgium, and the factors influencing the occurrence of domestic violence, we set up the study on relationships, stress, and aggression. In this study, we evaluate the prevalence of domestic violence victimization during the COVID-19 lockdown in Belgian children aged zero to seventeen years and the associations of the parents’ financial status, relationships, mental health, and previous victimization to the child’s victimization.

**Methods:**

A stepwise forward binary logistic regression was used to analyse the association between multiple risk factors of domestic violence and victimization of the respondent’s child. The respondent being an assailant, the respondent’s age, and the age of the children in the household were added as moderators.

**Results:**

In this model an association with domestic child abuse was found for the age of the respondent, the household’s size, the presence of children between zero and five years in the household, the perceived stress level of the respondent, and victimization of the respondent during the first wave of the sanitary measures, as well as victimization before the COVID-19 pandemic. None of the interacting effects were found to be significant.

**Conclusion:**

It is advisable to make extra efforts to improve well-being when maintaining sanitary measures by providing appropriate assistance and helping households struggling with increased or acute stress to install positive coping strategies - especially in larger households with children between six and 17 years. Besides, our findings draw attention to the clustering of risk of child and adult violence exposure in lockdown situations as well as to the potential cumulative impact of exposure to violence across the lifespan and across generations. It is key to invest in training healthcare workers and staff at schools to screen for and assess risks of domestic violence development and ongoing or past occurrence in order to detect, refer and follow-up on families at risk.

## Introduction

In March 2020, policymakers worldwide took measures to slow down and limit the spread of the COVID-19 virus. In Belgium, the Federal government announced a first lockdown with far-reaching isolation and movement-restricting measures on March 13, 2020. These sanitary measures were gradually and partly lifted over the summer of 2020. However, as the crisis continued to progress, a second wave of strict isolation and movement-restricting measures was put into place on November 1, 2020 [1].

While these measures were necessary to fight the spread of the virus, several experts warned for a significant impact on worse mental health and a potential increase in domestic violence [2–5]. Domestic violence comprises any behaviour in the family or domestic context, causing physical, psychological, sexual or socio-economic suffering to someone else [6]. Irrespective of whether victim(s) and assailant(s) share biological or legal family ties; the assailant(s) and victim(s) may (have) live(d) at the same residence. Domestic violence can thus also occur between both current and former (intimate) partners [7]. Domestic violence goes beyond intimate partner violence and also includes child, sibling, and elder abuse [8]. Domestic violence can engender a multitude of physical, psychological, sexual, social and economic consequences and may contribute to intergenerational transmission of violence leading to potential future violence [9–15] and is therefore an important public health problem [16].

The combination of a physical threat to health, unwanted and unexpected changes in financial and social stability, and the potential loss of loved ones in COVID-times, fuelled feelings of stress, anxiety, and frustration [17–19]. In many countries, including Belgium, an accumulation of negative feelings and an increase in conflicts within households resulted in an increase in reported domestic violence. These reports discussed existing domestic violence escalating further, but also mentioned the occurrence of violence in households where this had never happened before [20–24].

Besides the increase in children living in violent homes, an increase in children who were directly exposed to domestic violence and abuse was also reported [22]. Research on the multiple consequences of (in) direct domestic violence against children is well-established and suggests intergenerational transmission of violence, where people who have been directly or indirectly exposed to domestic violence during childhood are subsequently being revictimized or display aggressive behaviour themselves in adulthood, hence perpetuating the cycle of violence [22, 25, 26].

Additionally, the impact of the COVID-19 pandemic among families with respect to isolation and mental health in general was clearly demonstrated as well [22, 27]. Belgian’s federal research institute Sciensano organized a series of online surveys to monitor and evaluate the consequences of the pandemic. To estimate the impact of the crisis, the results of these online surveys, with a total sample of 17,774 Belgian respondents (18 years and older) [28], were compared with Belgian population data from before the COVID-19 pandemic [29]. According to this comparison, 16–24% reported anxiety and 14–22% reported depressive symptoms during the pandemic, compared to 11 and 9.5% respectively in 2018. Additionally, 10.5% reported suicidal thoughts in the past 12 months in June 2021, compared to only 4.3 in 2018 [28]. This decline in mental health in parents and in children due to the many isolation and movement-restricting measures, record levels of (temporary) unemployment, home schooling of children, financial instability, illness or death of relatives or even household members, the increase in children exposed to (in) direct violence and abuse, combined with the overall consequences of domestic violence, are likely to have an important impact on the children’s health, wellbeing and development in the long run.

On top of that, in Europe and also in Belgium, several domestic violence hotlines noticed an increase in the number of calls concerning violence [30–32], while Sexual Assault Care Centres reported less admissions during the strictest lockdowns and an increase when measures were lifted [33]. Victims reported additional barriers to escape problematic household situations during the lockdown and did not always manage to receive appropriate help in time [31, 33]. Social isolation, such as during lockdowns, is an especially important risk factor of abuse in children as research showed that all types of violence against children increased during school holidays and breaks [34, 35]. Besides, schools are for many children, a safe haven and the only option to find access to psychosocial support and mental health services through observative teachers or the school’s Student Guidance Centre [15, 22]. The forced closure of schools, therefore led to a decrease in reporting of child abuse during the pandemic and children did not manage to access the help they needed [36]. Failure to receive appropriate help in time can aggravate domestic violence as well as child abuse and fuel the risk of serious, long-term and sometimes life-threatening consequences in victims [37–39].

To study the impact of the COVID-19 measures in Belgium, and the factors influencing the occurrence of domestic violence in Belgium, we set up the study on “Relationships, Stress and Aggression in times of COVID-19” [40, 41]. The purpose of this paper, was to investigate the following aims: 1) evaluate the prevalence of domestic violence victimization during the second COVID-19 lockdown among Belgian children aged zero to seventeen years; and 2) examine the associations between the child’s victimization with the parents’ financial status, relationships, mental health and previous victimization.

## Material and Methods

### Study design and setting

This study builds on a previous study on Relationships, Stress and Aggression in times of COVID-19 in Belgium that was set up in March 2020 [20, 40, 42]. The current study on Relationships, Stress and Aggression in times of COVID-19 in Belgium [41] is based on a longitudinal online self-reported survey with two data collection waves, of which only the first wave of data collection will be used for this article. Respondents were able to participate to the first wave of data collection between 14 January and 28 February 2021. In that timeframe, we asked the respondents to reflect on their relationships, mental health and aggression experiences for the following three periods: 1) Before the sanitary measures (before March 13, 2020), 2) during the first wave of the sanitary measures (March 13, 2020 to the end of October 2020), and 3) during the second wave of the sanitary measures (1 November 2020 to the end of February 2021). The start of each wave corresponds with the start of new strict lockdown-measures. By the end of each wave, the measures were gradually and partly lifted, but never completely absent. The questionnaire was translated into Dutch, French, German, and English.

### Sampling and recruitment

Data were collected through an online survey via the survey software Qualtrics (Qualtrics, Provo, UT, USA). Only residents of Belgium who were 16 years or older at the time of participation were allowed to participate in the study. Participants were recruited through a variety of channels and methods, including press, social media, senior citizens’ organizations, mental health services, and snowball sampling where respondents were asked to further disseminate the questionnaire at the end of the survey. There were initially 4498 respondents, of which 2583 remained after exclusion of respondents who did not live in Belgium (*n* = 79), or did not complete the questionnaire (*n* = 1836).

### Measurement

The online survey contained questions regarding socio-demographics (age, gender, financial status and changes in the financial situation, level of education, household size, household composition and age of the children), sexual orientation, and gender identity, as well as satisfaction with physical encounters ‘How satisfied were you with the social contacts you had during face-to-face contacts’, online contacts ‘How satisfied were you with the social contacts you had online or by phone’, relationship with the partner ‘How satisfied have you generally been with the relationship’, and sexual life ‘How satisfied have you been with your sex life’ in the form of 12 five-point Likert scales (four per time period), ranging from ‘very dissatisfied (1)’ to ‘very satisfied (5)’. Respondents reporting no relationship could select ‘not applicable’ instead of filling in the Likert scales concerning the satisfaction with the relationship with the partner.

#### Acute stress symptoms

The prevalence of acute stress symptoms or Posttraumatic Stress Disorder (PTSD) was measured using the PC-PTSD-5 [43], which questioned symptoms in the month before completion of the questionnaire. On this scale with five items with a response format of ‘yes (1)/no (0)’ answers, a score of three of a maximum of five was regarded as an indication for PTSD [43].

#### Perceived stress

Perceived stress was measured by the Perceived Stress Scale (PSS) [44]. The scale had ten items, and responses were made on a five point Likert scale ranging from ‘Never (0)’ to ‘Very often (4)’. After rescaling half of the items to make sure they were all in the same direction (from most positive to most negative) all items were summed in a final score ranging from zero to 40 to yield a total perceived stress score, Cronbach’s Alpha = 0.900. The scale assessed symptoms in the month prior to filling in the survey and a cut-off of 14 was considered moderate stress and a cut-off of 27 was considered high perceived stress [44].

#### Depression and anxiety

Depression and anxiety were assessed using the Patient Health Questionnaire (PHQ)-4 [45]. This consists of a four-item four-point scale, ranging from ‘Not at all (0)’ to ‘Nearly every day (3)’ (Cronbach’s Alpha = 0.875) and assessing symptoms in the two weeks prior to filling in the survey. All items were summed in a final score ranging from zero to 12. Scores were rated as normal (0–2), mild (3–5), moderate (6–8), and severe (9–12).

#### Alcohol (ab)use

The AUDIT-C [46, 47] was used to assess alcohol (ab)use. The AUDIT-C consists of three questions, being ‘How often do you have a drink containing alcohol?’ ranging from ‘Never (0)’ to ‘4 or more times a week (4)’ (the screening ends with a score of 0 for respondents who indicated ‘Never’ in this first item), ‘How many standard drinks containing alcohol do you have on a typical day’ ranging from ‘1 or 2(0)’ to ‘10 or more (4)’ and ‘How often do you have six or more drinks on one occasion?’ ranging from ‘Never (0)’ to ‘Daily or almost daily (4)’. In accordance with the guidelines of the Flemish Expertise Center for Alcohol and Other Drugs (VAD)’, a cut-off score of four for females and five for males was used on this three item scale with a total score between zero and 12 [48].

#### Medication and drug (ab)use

In addition to the validated scales, the questionnaire also included yes-no questions about medication and drug use, suicidal thoughts, self-mutilation and suicide attempts before the start of the sanitary measures and during both waves.

#### Exposure to (in) direct violence

Violence was defined as forms of psychological, physical or sexual harm inflicted on another. The victimization questions of psychological and physical violence were based on previous research [37, 49]. A set of two yes/no questions were asked to assess direct psychological violence: ‘Did someone insult, criticize or belittle what you did or said’ and ‘Did someone do something to intimidate you’. Both items were recoded into the dichotomous variable ‘direct psychological violence’ with options ‘no (0)’ and ‘yes (1)’. Respondents reporting at least one ‘yes’ in the separate items would be coded as ‘yes’ in the new variable. The same methodology was applied to assess indirect psychological violence: ‘Do you know that someone in your household was insulted, criticized or belittled’ and ‘Do you know that someone else in your household was intimidated’ if the respondent indicated ‘My child or stepchild’ to the question ‘To whom did this happen’. Through these combinations of questions we were able to assess an approximate prevalence of violence on children. Direct physical violence was assessed in the exact same way with another set of two questions: ‘Did someone physically hurt or attack you’ and ‘Did someone (try to) stab, burn, maim, mutilate, strangle or kill you’ and repeated for indirect physical violence as well to assess the prevalence of physical violence on children.

A broad definition of sexual violence was used, taking into account both non-contact (hands-off) and physical (hands-on) forms, being in line with the current World Health Organization definition, starting from behaviour that is against one’s will [14, 50, 51]. The questions concerning sexual violence were based on previous research [14, 37] and multiple international validated questionnaires including the Sexual Experiences Survey [52], the National Intimate Partner and Sexual Violence Survey (NISVS) [53] and the Sexual Aggression and Victimization Scale (SAV-S) [54]. A set of six questions were asked to assess sexual victimization. The items can be grouped into two categories. Two items assessed hands-off sexual violence, including voyeurism and exhibitionism. The second category included four items on hands-on sexual victimization which can be further grouped into two sexual abuse items, including unwanted kissing and fondling/rubbing, and two attempted or completed rape items, including (attempted) oral, vaginal or anal penetration and being forced to penetrate someone else. The questions were asked in the same way as for psychological and physical violence and were recoded in the exact same manner for both direct and indirect sexual violence.

Finally, a total score was also computed, where respondents reporting at least one ‘yes’ in any of the ten previously discussed items concerning direct victimization would be coded as ‘yes (1)’ in the variable ‘victimization’. If the respondents reported at least one ‘yes’ in any of the ten items concerning indirect victimization which concerned their (step) child, it would be coded as ‘yes (1)’ in the variable ‘victimization of the respondent’s child’.

Participants were also asked to indicate the person who did this to them or to their child. This question was asked for each of the above items separately for direct and indirect exposure to violence.

### Statistical analysis

Data was imported into SPSS27 for initial data cleaning and data manipulation. All statistical analyses were conducted with R software version 4.0.3. Simple descriptive statistics were analysed and group differences in the outcome variable were computed using a chi-square test or Fisher’s exact test if the assumptions of the chi-square test could not be met. A stepwise forward binary logistic regression was used to analyse the association between the multiple factors and the outcome variable. A large amount of potential predictor variables is known to correlate with child abuse. However a stepwise regression allows us to identify the best predictor variables from all the available options. The interacting effects of the respondent being an assailant (yes or no) of violence, the respondent’s age (three categories) and the age of the children in the household (three different variables) in the relationship between the predictor variables and child abuse were also added to the model. To avoid multicollinearity, the correlations were checked between all variables before proceeding to model building. During the model building the multicollinearity assumption of multivariate regression analyses was also tested for the main effects with the VIF and indicated no violation. Main and interacting terms with *p* < 0.05 were included in the model, and variables that produced at least one beta estimate significantly different from zero were retained. It was also determined whether these added main and interacting effects significantly improved the prediction of the outcome variable using a likelihood ratio test. Besides, the Akaike Information Criterion (AIC) was used to compare the relative quality of one model to another by balancing a model’s goodness-of-fit against its complexity. In other words, it takes into account the risk of overfitting as well as the risk of underfitting. Models were then ranked from best to worst with the “best” model showing the smallest AIC. Binary logistic regression was chosen because the outcome variable consisted of two categories (the respondent has no child being a victim of violence during the second wave of sanitary measures = 0, the respondent has at least one child being a victim of violence during the second wave of sanitary measures = 1). Finally, the odds ratios were calculated with their 95% confidence intervals (CI).

## Results

Respondents who did not complete at least one of the questions on domestic violence were excluded from the analysis. The descriptive statistics of the respondents and their child (ren) can be found in Table 1. The participants were mainly female (81%) and had a mean age of 40 years (CI: 33.42–47.44). A large majority (81%) finished higher education. One in four respondents (25%) reported a more difficult financial situation since the first wave of sanitary measures and one in five (19%) during the second wave.

**Table 1:** Descriptive statistics of the respondents and their child (ren)

	n (***n*** = 870)	%	% children victimized	X^**2**^; df; ***p***-value
**Age** [mean = 40.43; SD = 7.01]				15.14; 3; **.002**
22–34 years	179	20.57	7.26	
35–44 years	476	54.71	16.18	
45–54 years	184	21.15	21.74	
> 54 years	31	3.56	12.90	
**Gender**				.110*
Female (incl. trans-women)	706	81.15	16.43	
Male (incl. trans-men)	160	18.39	10.62	
Other	3	0.34	33.33	
**Education**				.991*
No school or primary school	4	0.46	25.00	
Highschool (technical, religious, …)	158	18.16	15.19	
Higher education (University, College, …)	708	81.38	15.40	
**Financial difficulty 1st wave**				1.38; 2; .501
No	643	73.91	15.55	
Yes	216	24.83	14.35	
No answer	11	1.26	27.27	
**Financial difficulty 2nd wave**				.86; 2; .650
No	693	79.65	15.30	
Yes	165	18.96	15.15	
No answer	12	1.38	25.00	
**Household size**^a^ [mean = 2.77; SD = 0.95]				13.78; 4; .008
1	67	7.70	16.42	
2	260	29.88	11.54	
3	389	44.72	14.14	
4	124	14.25	24.19	
≥ 5	30	3.45	26.67	
**Age of the children**^b^				
0–5 years	401	46.09	9.98	16.81; 1; **<.001**
6–12 years	500	57.47	19.20	13.01; 1; **<.001**
13-17 years	262	30.11	24.05	21.50; 1; **<.001**

^a^Respondents are not included in the count. 1 = the respondent lives with another person in the same household. In this case 1 = the respondent lives with 1 child. 2 = the respondent lives with 1 child and 1 other adult (e.g. partner) or with 2 children. 3 = the respondent lives with 1 to 3 children (if 2 children, then 1 adult or if 1 child, then 2 adults, …

^b^Respondents were able to select multiple age-categories for their children, which means that the total percentage of these three age-categories can surpass 100%. Respondents with children aged older than 17 years were excluded from the sample

* Fisher’s Exact Test (instead of Chi Square Test): *p*-value

Because the comparisons in this table involved 9 independent tests, we adopted a Bonferroni-corrected significance level of .05/9 = .006 for these analyses

*Abbreviations*: *SD* Standard deviation

One in three (33.68%) or 870 respondents had at least one child and all children of the household were younger than 18 years. In addition, 8 % lived alone with one child, 30% lived with two other members in their household (this could be two children or one adult, such as for example the partner of the respondent, and one child), 45% with three, 14% with four and 3 % with five or more. Almost half (46%) of the respondents had at least one child younger than six years, 57% had at least one child between six and 12 years and one in three (30%) had a child between 13 and 17 years.

Table 1 shows the distribution of the different variables examined as well as the presence of domestic violence in children in the different values of the variables (fourth column). From the last column can be concluded that the age of the respondent (parent) and the age of the respondent’s child (ren) had a significant difference in the distribution of domestic violence against children within the different values of the two variables. For example, the youngest group (22–34 years) had a significantly lower proportion of victimized children than the older group (45–54 years). Finally, there were proportionally more victimized children in families with at least one child between the age of six and 17 years. In the case of households with at least one child between the age of zero to five the proportion of victimized children decreased instead.

While respondents were mostly (very) satisfied with their physical (92%) and online/telephonic (69%) contacts before the COVID-19 pandemic, the satisfaction rate decreased dramatically to 21% during the first wave of sanitary measures and even further to 15% during the second wave for physical encounters. The same trend was seen for online/telephonic contacts with 55 and 44%, respectively. Respondents were mostly (very) satisfied (76%) with the relationship they had with their partner and their sexual life (68%) before the COVID-19 pandemic. These positive satisfaction rates remained high, but decreased slightly to 69% during the first wave and 64% during the second wave for the relationship with the partner and to 57% during the first wave and 53% during the second wave when considering their sexual life. There were no significant differences in satisfaction distribution between households with or without a victimized child.

However, significant differences in mental health were found between households with or without a victimized child and can be found in Table 2. The prevalence of ASS was higher in households with a victimized child and respondents reporting domestic child abuse had proportionally higher rates of perceived stress and psychological distress during the COVID-19 pandemic. One in five respondents (21%) had three or more acute stress symptoms, hinting to the presence of PTSD. Further, 62% reported moderate stress and 15% reported high stress rates. One in three respondents reported moderate to high psychological distress in the form of anxiety and depressive symptoms. Additionally, high rates of problematic alcohol intake were reported for the period before the pandemic (51%) and during the pandemic (50%). There was however no significant association of alcohol intake with the prevalence of domestic child abuse.

**Table 2:** Descriptive statistics on the different variables surrounding mental health and coping mechanisms

	Before the COVID-19 pandemic			During the COVID-19 pandemic^a^		
	n (%)	% children victimized	X^2^; df; *p*-value	n (%)	% children victimized	X^2^; df; *p*-value
**Mental health**						
Acute stress symptoms (ASS)	–	–	–			20.85; 1; **<.001**
No				687 (78.97)	12.52	
Yes				183 (21.03)	26.23	
Perceived stress (PSS)	–	–	–			21.67; 2; **<.001**
Low				195 (22.41)	8.21	
Moderate				542 (62.30)	15.13	
High				133 (15.29)	27.07	
Psychological distress (PHQ-4)	–	–	–			28.87; 3; **<.001**
No				272 (31.26)	9.56	
Mild				315 (36.21)	14.60	
Moderate				158 (18.16)	15.19	
Severe				125 (14.37)	30.40	
**(Ab) use of**						
Alcohol			4.27; 1; .039			0.32; 1; .573
No	422 (48.51)	18.01		435 (50.00)	14.71	
Problematic	448 (51.49)	12.95		435 (50.00)	16.09	
Sedatives			14.01; 2; **<.001**			13.93; 2; **<.001**
No	547 (62.87)	11.88		638 (73.33)	12.62	
Yes	314 (36.09)	21.34		228 (26.12)	22.81	
No answer	9 (1.03)	22.22		8 (.92)	25.00	
THC			1.575; 2; .455			2.42; 2; .298
No	644 (74.02)	15.68		815 (93.68)	15.09	
Yes	215 (24.71)	13.95		46 (5.29)	17.39	
No answer	11 (1.26)	27.27		9 (1.03)	33.33	
Stimulants			0.511; 2; .774			.161*
No	791 (90.92)	15.42		844 (97.01)	15.17	
Yes	65 (7.47)	13.85		15 (1.72)	13.33	
No answer	14 (1.61)	21.43		11 (1.26)	36.36	
**Self-harming behaviour & suicidal ideation**						
Suicidal thoughts			24.00; 2; **<.001**			33.09; 2; **<.001**
No	591 (67.93)	11.34		706 (81.15)	12.04	
Yes	257 (29.54)	24.51		147 (16.90)	30.61	
No answer	22 (2.53)	18.18		17 (1.95)	23.53	
Suicide attempts			8.01; 2; .018			1.000*
No	815 (93.68)	14.72		858 (98.62)	15.50	
Yes	43 (4.94)	30.23		2 (.23)	0	
No answer	12 (1.38)	8.33		10 (1.15)	10.00	
Self-mutilation			9.80; 2; .007			12.17; 2; **.002**
No	777 (89.31)	14.29		841 (96.67)	14.86	
Yes	81 (9.31)	27.16		18 (2.07)	44.44	
No answer	12 (1.38)	8.33		11 (1.26)	9.09	

^a^During the COVID-19 pandemic: Only the second wave for the mental health variables. The first and second wave were taken together for the (ab) use and self-harming behaviour and suicidal ideation variables

* Fisher’s Exact Test (instead of Chi Square Test): *p*-value

A corrected p-level of .05/7 = .007 was used as the critical significance level for the 1st set of comparisons (before the COVID-19 pandemic). A corrected p-level of .05/10 = .005 was used as the critical significance level for the 2nd set of comparisons (during the COVID-19 pandemic). The mental health scales were not asked for the period before the start of the COVID-19 pandemic

In total, one in three respondents indicated that they had been directly exposed to violence during the first (33%) and/or second (34%) wave of the sanitary measures (Table 3). One in four (27%) were victimized during both waves. Sixty-one percent of the respondents experienced violence at some point before the first COVID-19 lockdown. Respondents who were directly exposed to violence had a significantly higher prevalence of children that were exposed to violence as well, except in the case of sexual violence before the COVID-19-pandemic and sexual violence during the second wave of the sanitary measures.

**Table 3:** Descriptive statistics on the prevalence of violence (psychological, physical and sexual) concerning the respondent

	Before the COVID-19 pandemic			During the 1st wave of the pandemic			During the 2nd wave of the pandemic		
	n (%)	% children victimized	X^2^; df; *p*-value	n (%)	% children victimized	X^2^; df; *p*-value	n (%)	% children victimized	X^2^; df; *p*-value
**Respondent as victim of**									
Psychological violence			48.63; 1; **<.001**			37.86; 1; **<.001**			47.59; 1; **<.001**
No	389 (44.71)	5.91		594 (68.28)	10.27		587 (67.47)	9.54	
Yes	481 (55.29)	23.08		276 (31.72)	26.45		283 (32.53)	27.56	
Physical violence			18.91; 1; **<.001**			27.11; 1; **<.001**			31.35; 1; **<.001**
No	696 (80.00)	12.97		830 (95.40)	14.29		836 (96.09)	14.30	
Yes	174 (20.00)	26.44		40 (4.60)	45.00		34 (3.91)	50.00	
Sexual violence			2.94; 1; .086			10.83; 1; **<.001**			2.95; 1; .086
No	658 (75.63)	14.80		845 (97.13)	15.34		841 (96.67)	15.66	
Yes	212 (24.37)	19.81		25 (2.87)	40.00		29 (3.33)	27.59	
Total violence			41.93; 1; **<.001**			39.42; 1; **<.001**			46.53; 1; **<.001**
No	342 (39.31)	5.56		581 (66.78)	9.98		574 (65.98)	9.41	
Yes	528 (60.69)	21.78		289 (33.22)	26.30		296 (34.02)	27.03	

Fourteen percent of the respondents (*n* = 118) in this sample had at least one child being exposed to psychological violence and 5 % (*n* = 40) to physical violence during the second wave of the sanitary measures. Only two children were exposed to (hands-on) sexual violence (without penetration). A total of 134 respondents or 15% of the sample reported at least one victimized child in their household during the second wave of the COVID-19 pandemic. The assailants were mainly part of the household with 28% being the respondent him or herself (parent of the child), 35% being the (ex-)partner of the respondent and 10% being a sibling of the victimized child. Only two respondents reported the grandparents of the child as the assailant and 1 respondent reported another person of the household, but being not part of the family. Forty-two percent of the respondents with a victimized child reported an assailant that was not part of the household. Respondents were able to select multiple assailants across different forms of violence, which means that the total percentage of the assailant’s categories surpasses 100%. Finally, 63% of the respondents with a victimized child reported a male assailant and 45% reported a female assailant.

The final model (Table 4), with the highest predictive value, contains the age of the respondent, the household’s size, the presence of children between zero and five years in the household, the perceived stress level of the respondent and victimization of the respondent during the first wave of the sanitary measures, as well as victimization before the COVID-19 pandemic. The C-index of 0.76 shows a useful and satisfying predictive value of the model and corresponds to the area under the ROC curve. In other words, victimization of the respondent’s child (ren) would be correctly predicted for 76% of the respondents if the model below was used. The explained variance of the model equals 26%. Victimization of the respondent during the second wave was also found to be significantly correlated to child victimization, but could not be kept in the model due to multicollinearity with victimization during the first wave.

**Table 4:** Predictors for the victimization of the respondent’s child during the second wave of sanitary measures”

Predictors	Descriptives (%)	Estimate	EXP(B) Odds ratio	95% C. I . Odds ratio	***p***
**Age of the respondent** (ref. 22–34 years)	20.57				**<.001**
35–44 years	54.71	0.524	1.689	0.866–3.478	
45–54 years	21.15	0.723	2.062	0.931–4.742	
> 54 years	3.56	0.432	1.540	0.368–5.500	
**Household with child (ren) aged 0 to 5**^a^ (ref. No)	53.91				**.004**
Yes	46.09	−0.707	0.493	0.299–0.801	
**Household size** (ref. 2)	29.88				**.023**
1	7.70	0.017	1.017	0.448–2.181	
3	44.72	0.370	1.447	0.877–2.429	
4	14.25	0.993	2.699	1.474–4.963	
5 and more	3.45	1.156	3.177	1.167–8.112	
**Perceived stress** (ref. Low)	22.41				**<.001**
Moderate	62.30	0.406	1.501	0.844–2.805	
High	15.29	0.903	2.467	1.242–5.059	
**Victimization**^b^ **before** (ref. No)	39.31				**<.001**
Yes	60.69	1.251	3.494	2.024–6.275	
**Victimization**^b^ **first wave** (ref. No)	66.78				**.009**
Yes	33.22	0.542	1.719	1.112–2.700	

^a^These households can also have children that are between 6 and 17 years old, but have at least one child between 0 and 5

^b^Respondents (parent of the child) as victim of violence (psychological, physical and/or sexual)

*Abbreviations*: *C.I* Confidence Interval

The odds ratio show that, if all other variables remain equal, the risk of a child being a victim of violence was 2.7 times higher for respondents living with another four household members and 3.2 times higher for respondents living with five or more household members compared to respondents living with only two additional household members. The risk of a respondent having a victimized child was also half as high for respondents with at least one child between the age of zero and five years (regardless of whether or not this is the victimized child or their sibling(s)), compared to respondents who had only children older than six years. Respondents with a high perceived stress level had a 2.5 times higher risk of having a child being a victim of violence than respondents who had a low perceived stress level. There was no significant difference between respondents with low or moderate stress levels. Finally, the risk of a child being a victim of violence was 3.5 times higher for respondents who were a victim of violence themselves before the COVID-19-pandemic and 1.7 times higher for respondents who were a victim of violence during the first wave of the sanitary measures compared to respondents who were never a victim of violence. There was no significant moderating effect of the respondent being an assailant (yes or no) of violence themselves, the age of the respondents, and the age of the children in the household. This also means that there is no evidence that the age of the respondents is connected to the age of the children which in turn might influence the prevalence of victimized children.

## Discussion

Since the COVID-19 pandemic, many experts feared an increase in domestic violence and child abuse due to the isolation and movement-restricting measures [3, 32, 34, 36]. In the general study on Relationships, Stress and Aggression in times of COVID-19 in Belgium, one in four respondents reported domestic violence since the onset of the COVID-19 pandemic (25% during the first wave and 24% during the second wave) [41]. The study in this paper focusses on a subsample of this general study, namely households with at least one child and all children under the age of 18 years. In this subsample the prevalence is even higher with one in three respondents indicating that they themselves had become victim of domestic violence (33% during the first wave and 34% during the second wave), and 15% of the respondents reporting to have witnessed at least one child in their households to have been victimized during the second wave of COVID-19 related sanitary measures. With regard to victimized children, this study additionally shows that it is not so much the amount of time spent at home, the educational level of the respondent or the financial situation of the household, but rather the increased stress of the parent and a history of violence in the household that is associated with an increased risk of victimization in the children. Although it cannot be deduced from this study whether the increase in stress level is a direct result of the direct consequence of the lockdown measures, a few studies during the COVID-19 pandemic did show an increase in symptoms of anxiety, depression and stress during the same lockdown periods as this study compared to the years before the pandemic [28, 55]. From this we could infer that the lockdown measures, which led to isolation, record levels of (temporary) unemployment, home schooling of children, financial instability, illness or death of relatives or even household members, and so on, are causing increased stress, which in turn can contribute to a higher risk of domestic violence. It remains however unsure if stress is a risk factor of violence, a consequences of violence exposure, or both.

Despite the fact that the direction of the association between stress and violence is not yet entirely clear, our results suggest the intrinsic link between violence and stress. Previous research has already shown that psychological frailty and stress were risk factors for (re) victimization and perpetration of violence in general, but also of domestic violence [56–58]. Moreover, extensive research also showed that domestic violence can have particularly important consequences for the mental health of victims of all genders [13, 57, 59, 60]. In our study, an increased perceived stress level of the respondent seemed an important predictor for victimization of the respondent’s child, which is in line with previous research where parents in stressful life circumstances were found to be positively associated with perpetration of child abuse [58]. We therefore think it is advisable to make extra efforts to improve well-being when maintaining sanitary measures by providing appropriate assistance and helping households struggling with increased or acute stress to install positive coping strategies.

In addition, we found that a history of any form of violence experienced by the respondent (psychological, physical, and/or sexual) before the COVID-19 pandemic or during the first wave, also increased the risk of child victimization during the second wave. This confirms previous research where the prevalence of child abuse was found to be consistently higher for parents who reported traumatic events themselves, such as sexual victimization, but also other forms of domestic violence [9]. Moreover, the occurrence of violence experienced by the respondent during the second wave was also correlated to the occurrence of child abuse. One in five respondents (27%) who were victimized themselves during the second wave reported a victimized child, compared to only one in ten respondents (9%) if they were not victimized themselves. In 28% of the cases the respondent was the assailant him or herself (parent of the child) and in 35% of the cases the (ex-)partner of the respondent (parent or step-parent of the child) leading to more than half of the children being victimized by their own (step-)parents. Additionally, 10% of the children were victimized by their siblings, living in the same household. All of this points in the direction of a complex web of domestic violence and potential intergenerational violence [10, 11, 61].

In conclusion, our findings draw attention to the clustering of risk of child and adult violence exposure in lockdown situations as well as to the potential cumulative impact of exposure to violence across one’s lifetime and across generations. First of all, given the found link between stress and domestic violence, it is important that leisure activities that help family members to maintain and improve their mental health including coping with and reducing stress, are encouraged in family or household health in all promotion programs, regardless of any future lockdown measures. Next, it is key to invest in training healthcare workers to screen for and assess risks of domestic violence development and ongoing or past occurrence in order to detect, refer and follow-up on people at risk. Therapists and health care practitioners who, for example, work with individuals or couples who report high levels of stress and/or abuse should routinely assess for domestic violence in general and consider ways to expand the treatment to include children as well. In addition to applying this in settings of general practitioners and hospitals, it might be wise to implement this at low threshold health services such as COVID-19 testing and vaccination sites, on the condition that they are able to provide follow-up care and the right referrals if needed. For households with children, staff at schools and their Student Guidance Centre could also be provided with adequate tools and trainings. Such tools and trainings concerning domestic violence are highly needed for professionals working with children, regardless of the presence of a pandemic. During the past two years a lot of online alternatives came up, making it possible to help children even in the case of future movement restricting measures. Finally, policy makers should make prevention of and response to domestic violence a priority in the action plan containing for the impact of COVID-19 sanitary measures and recovery.

### Limitations

Our study has several limitations. First, despite the satisfying number of respondents, the main limitation of this study concerns selection bias. Given the sampling method, our research results, cannot simply be generalized to the Belgian population. Women and more highly educated people were overrepresented in the sample. It is likely that we underestimate the proportion of those with a more vulnerable socioeconomic status. The effect of this selection bias is however unclear. However, the higher proportion of women makes it possible that the proportion of female assailants was underestimated and the proportion of male assailants was overestimated. Second, the prevalence of child abuse is very likely to be underestimated as we could only draw conclusions from the reported indirect violence experiences of the respondent, leaving out cases of child abuse with no witnesses to report about them. Third, the retrospective survey of violence, which may induce a reporting bias, also limits the generalizability of our results. Participants were asked to complete questions over three different points in time, which might lead to inconsistencies as the first two periods might not be well remembered or even distorted, leading to a memory of events that might differ from reality. A prospective study would have further strengthened our conclusions. Fourth, a broad definition of sexual violence, including both hands-off and hands-on sexual violence was used. In the regression we went even further by combining all forms of violence (psychological, physical and sexual) together. One might assume that these different forms of violence might have a different impact on child abuse. However, due to high levels of multicollinearity (VIF > 4) and all forms of violence being each separately associated to child abuse, we decided to combine these forms into a general domestic violence variable. Finally, a different model could have been fit for each form of child abuse (psychological, physical, sexual), but was not possible due to the small sample of children that were reported to have been physically or sexually abused.

## Conclusion

In this model an association with domestic child abuse was found for the age of the respondent, the household’s size, the presence of children between 0 and 5 years in the household, the perceived stress level of the respondent, and victimization of the respondent during the first wave of the sanitary measures, as well as victimization before the COVID-19 pandemic. It therefore seems appropriate to include these results in the prevention and response to domestic violence in comparable lockdown situations. Given that domestic violence has an impact on the victims, assailants and people who are indirectly exposed to it, it seems crucial to focus on prevention and quality care for all of the people involved, and thus the household as a whole.

## Data Availability

We are unable to make our data set publicly available for ethical reasons. This study involves sensitive human research participant data, which cannot be shared publicly. However, the corresponding author can be contacted for future data request purposes (may require data use agreements to be developed).
